# Induction of VX2 para-renal carcinoma in rabbits: generation of animal model for loco-regional treatments of solid tumors

**DOI:** 10.1186/s13027-016-0103-8

**Published:** 2016-12-01

**Authors:** Sabrina Bimonte, Maddalena Leongito, Mauro Piccirillo, Maria Luisa Tamma, Marianna Vallifuoco, Adele Bracco, Antonio Mancini, Daniele Di Napoli, Sigismondo Castaldo, Santolo Cozzolino, Francesca Iacobellis, Roberto Grassi, Vincenza Granata, Secondo Lastoria, Steven Curley, Francesco Izzo

**Affiliations:** 1 Division of Abdominal Surgical Oncology, Hepatobiliary Unit, Istituto Nazionale per lo studio e la cura dei Tumori “Fondazione G. Pascale”, IRCCS, Via Mariano Semmola, 80131 Naples, Italy; 2 Center of Biotechnologies, Antonio Cardarelli Hospital, Naples, Italy; 3 Institute of Radiology, Second University of Naples, Naples, Italy; 4 Division of Radiology, Istituto Nazionale per lo studio e la cura dei Tumori “Fondazione G. Pascale”,-IRCCS, Naples, Italy; 5 Department of Diagnostic Imaging and Radiotherapy, Division of Nuclear Medicine, Istituto Nazionale Tumori “Fondazione G.Pascale” - IRCCS, Naples, Italy; 6 Department of Surgery, Baylor College of Medicine, One Baylor Plaza, Houston, TX 77030 USA

**Keywords:** Kidney, Rabbits, VX2, Para-renal neoplasm

## Abstract

**Background:**

Animal models of para-renal cancer can provide useful information for the evaluation of tumor response to loco-regional therapy experiments in solid tumors. The aim of our study was to establish a rabbit para-renal cancer model using locally implanted VX2 tumors.

**Methods:**

In order to generate a rabbit model of para-renal cancer, we established four hind limb donor rabbits by using frozen VX2 tumor samples. Following inoculation, rabbits were monitored for appetite and signs of pain. Viable tumors appeared as palpable nodules within 2 weeks of inoculation. Tumor growth was confirmed in all rabbits by high-resolution ultrasound analysis and histology. Once tumor growth was established, hind limb tumors extraction was used for tumor line propagation and para-renal tumor creation. Twenty-one rabbit models bearing para-renal cancer were established by implanting VX2 tumor into the para-renal capsula. Tumors developed into discreet 2–3 cm nodules within 1–3 weeks of implantation. Serial renal ultrasonography follow-up, starting 1 week after tumor implantation, was performed. Two weeks after tumor implantation, rabbits were euthanized and tumors and other organs were collected for histopathology.

**Results:**

Tumor growth after VX2 tumor fragment implantation was confirmed in all rabbits by high-resolution ultrasound (US) imaging examinations of the para-renal regions and was measured with digital caliper. The para-renal injection of VX2 tumor fragments, achieved tumor growth in 100% of cases. All data were confirmed by histological analysis.

**Conclusions:**

We generated for the first time, a model of para-renal cancer by surgical tumor implantation of VX2 frozen tumor fragments into rabbit’s para-renal region. This method minimizes the development of metastases and the use of non-necrotic tumors and will optimize the evaluation of tumor response to loco-regional therapy experiments.

## Background

The rabbit VX2 tumor model is frequently used in experimental oncology. The VX2 tumor is a leporine anaplastic squamous cell carcinoma induced by virus. Hypervascularity, rapid growth and easy propagation in the skeletal muscle, represent its features [1–4]. It is of note that this model has been used to model various types of cancer, including those of pancreas, kidney and liver [5–15]. The transplantation of VX2 cells can be achieved either by implanting solid tumor pieces or by injecting the tumor cell suspensions (fresh or frozen) as previously described [16].

Various form of multimodal neoadjuvant and adjuvant treatments have been used in combination with surgery to improve loco regional control of solid tumors having a high incidence of local recurrence after resection, such as rectal cancer [17–21], soft tissue sarcomas [22] and pancreatic cancer [23, 24]. The present study describes the establishment of a rabbit para-renal cancer model using locally implanted VX2 frozen tumors. This cancer model will be potentially used for the evaluation of tumor response to multimodal treatment of solid tumors with high risk of local recurrence after surgery.

## Methods

### Animal preparation

All experiments were carried out with the approval of the Ethics Committee and the Italian Ministry of Health, and the professional staff of the biotechnologies animal research facility supervised the housing and care of the rabbits. Twenty-five Male New Zealand White rabbits (body weight of 2.5–3.5 kg) were initially fed with standard chow and water ad libitum for a week during adaptation in the animal research facility. After all the surgical procedures and the treatments established by the experimental protocol, the rabbits were placed in a cage temporarily heated for the recovery of vital functions and then were transferred to regular cages. All procedures were performed in a sterile environment.

### VX2 tumor fragments preparation

VX2 tumor fragments (obtained by Prof. Agata Exner from Case Western Reserve University Cleveland Ohio) were stored in freeze media at – 80 °C until first use. The vials containing tumor fragments were placed in a 37 °C water bath for a rapid but not complete thawing. Tumor pieces were removed carefully from the cryotubes with a sterile anatomic surgical forceps and placed into a sterile Petri dish 100 mm × 20 mm (Corning Inc.,) in ice. The medium contained 25 ml Dulbecco’s Modified Eagle Medium/nutrient mixture F-12 with glutamine (DMEM/F12, Aurogene) and 5% Fetal Bovine Serum (FBS, Sigma-Aldrich). Subsequently, the fragments were also washed in cold Hanks’ Balanced Salt Solution (HBSS Euroclone). In both washing procedures, the pieces were cleaned carefully 3 times. The tumor bits were placed into a sterile Petri dish with 15 mL of cold HBSS and were completely covered by ice until implantation in animals.

### VX2 hind limb tumor development and harvesting

Four NWZ rabbits were used to propagate the VX2 tumor in lower para-spinal muscles. All rabbits were pre-anesthetized with a combination of medetomidine (0.2 mg/kg), ketamine (10 mg/kg) and butorphanol (0.5 mg/kg) pre-operatively. General anesthesia was induced through oral endotracheal intubation with a fibre-optic scope and was maintained with sevorane 2% and ketamine hydrochloride (30 mg/kg i.v.). After anesthesia and pre-operative skin preparation, an incision of 2.5 cm was made on the right hind limb until reaching the muscle belly. Two small tumor pieces of 5 mm^3^ each were implanted into the quadriceps muscle of the hind limb and the gap was sutured with prolene 4/0. Hemostasis was confirmed and closure in layers with interrupted sutures (vycril 2/0), was performed. A viable tumor is evident as a palpable nodule of about 2,5 cm × 3 cm within 10–14 days after tumor implantation. Tumor growth was confirmed by ultrasonography and then hind limb tumor extraction was performed. Each rabbit, under general anesthesia received a lethal injection of Tanax 1 ml/Kg, at sacrifice. Subsequently, skin preparation and an incision of 4–5 cm were made on the right hind limb until reaching the surrounding tumor area. Two curvilinear incisions were made within the muscle, in order to remove the tumor mass en-bloc as described in Parvivian et al.[16]. The explanted tumor was bisected in order to detect any gross evidence of a necrotic core to obtain viable peripheral tumor. The resected tumor masses were put in a sterile 50 ml tube containing ~30 ml serum free RPMI 1640, (Aurogene) with 3% Penicillin 10,000 IU/ml, Streptomycin 10,000 ug/ml (Aurogene) and 1% Amphotericin B, 250 ug/ml (Sigma-Aldrich) for 30 min. Tumor tissues were placed into a 100 mm Petri dish, and any visible non-tumor tissue, was trimmed away. The tissue was minced into several pieces (about 5 mm^3^ each), using a sterile scalpel (#10) and forceps. Tumor pieces were rapidly washed in ~20 ml HBSS, ~20 ml DMEM/F12 plus 5% FBS, 3 times each. Half of tumor tissues were employed fresh to develop a para-renal tumor model, while the remaining part was frozen to create a future stock. Freezing procedure was performed by placing the tumor pieces into cry vials of 5 ml containing ~3 ml of freeze media 70% DMEM/F12, 20% FBS and 10% Diethyl Sulfoxide (DMSO, Fisher Scientific). Cryovials were placed in ice for 30 min, transferred at −20 °C for 2 h and then stored at – 80 °C.

### Generation of VX2 para-renal tumor rabbit model

Twenty-one rabbits were used in this study, five of them were implanted with fresh tumor tissues, while sixteen with frozen tumor pieces. Immediately before the sacrifice of animals, we obtained samples of blood by aspiration with a 24 guage needle from the rabbits ears.

The blood samples were centrifuged (3000 rpm, 5 min) to isolate the plasma fraction. Baseline, and liver enzyme (ALT, AST, GGT, Alkaline phosphatase) bilirubin and Complete Blood Counts (CBC) were evaluated and repeated every 7 days, until the sacrifice of animals. For the para-renal tumor implantation procedure, the recipient rabbits received xipho-umbilical laparotomy. A blunt dissection was used to expose the peritoneum through the avascular linea alba. Careful division of the peritoneum allows exploring the peritoneal cavity. Intestinal mobilization was performed and an incision of the left retroperitoneal space supra renal capsule was made. A single tumor fragment was then placed in this para-renal space. The incision was closed with prolene 4/0. After confirmation of hemeostasis, the abdominal wall incision was closed (peritoneum and fascia with Vycril 2/0 in continuous suture, the skin in interrupted sutures in silk 2/0). After all the surgical procedures and the treatments established by the experimental protocol, the rabbits were placed in a cage temporarily heated for the recovery of vital functions and then were transferred to regular cages. Daily observations were made regarding appetite, feces and urine quantity and consistency, attitude and activity levels. Appropriate treatment measures were employed to address any abnormalities noted during the daily observation and recorded in appropriate animal welfare sheets. Tumor growth was assessed by in vivo ultrasound imaging.

### In *vivo* high-resolution ultrasound imaging

High-resolution ultrasound (US) imaging examinations were performed with a VisualSonicsVevo 2100 unit (Visual Sonics Inc., Toronto, Canada) specifically customized for animal research. Abdominal radiologist (F.I.) with 5 years of experience in radiology and in small animal imaging field, performed all the micro-US examinations. Each rabbit was positioned on a dedicated table in supine position. Body temperature was monitored by a rectal probe and maintained at 37.0 ± 0.5 °C with a heating blanket regulated by a homoeothermic blanket control unit of which the US unit is equipped. An appropriate amount of heated sonographic gel (Aquasonic 100; Parker Laboratories, Inc, Fairfield, NJ) was applied on the abdomen. Images were acquired by using a linear array (MS250D, Visual Sonics; frequency 13–24 MHz), fixed on an appropriate arm to ensure a stationary position and constant pressure on the desired image plane. US examination was performed in B-mode, with the following setting parameters: frequency, 21 MHz; dynamic range, 65 dB; frame rate, 8; depth, 21 mm; width, 23 mm; transmit power, 100%; high line density. All imaging settings were kept constant throughout imaging sessions for all animals. Animals were examined on the 7th and the 14th day after surgery focusing on the development of heteroplastic tissue in the site of the surgical implant and on the presence of hepatic metastases. In each rabbit, micro-US was performed exploring all the abdominal parenchymatous organs and the peritoneal recesses in order to evaluate the presence of any parenchymal lesion or intra-retroperitoneal free fluid. Particularly, the size, shape, and location of the kidneys were detected and the peri and para renal spaces were explored. When presence of new heteroplastic masses was detected, their size and shape were assessed in the three orthogonal planes.

### Histological examination

The animals were euthanized under general anesthesia; the kidneys were harvested and examined macroscopically and microscopically. Each organ was sliced into 5-mm slices, and the malignant lesions were determined by the consensus of two observers (L.M. and F.T.). Representative samples were frozen, sectioned in a cryostat at 5-lm thickness, and stained using routine hematoxylin and eosin for light microscopy.

## Results

### Generation of VX2 limb tumor rabbit model

**Fig. 1:**
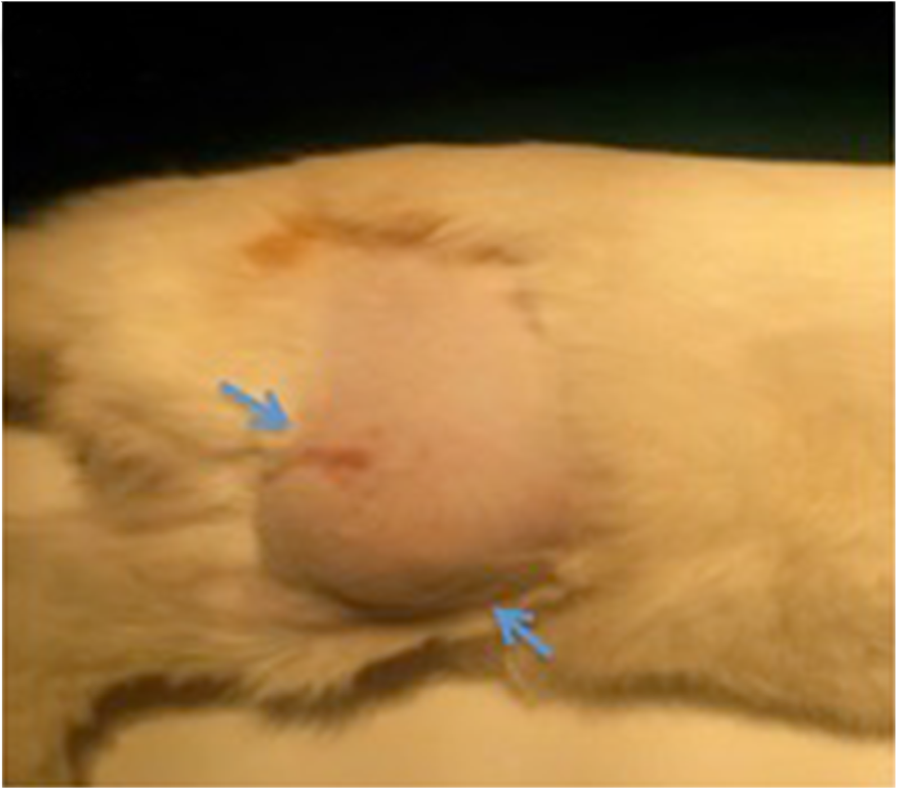
Hind limb VX2 tumor development. Picture reveals visible subcutaneous hind limb tumors (*arrows*)

**Fig. 2:**
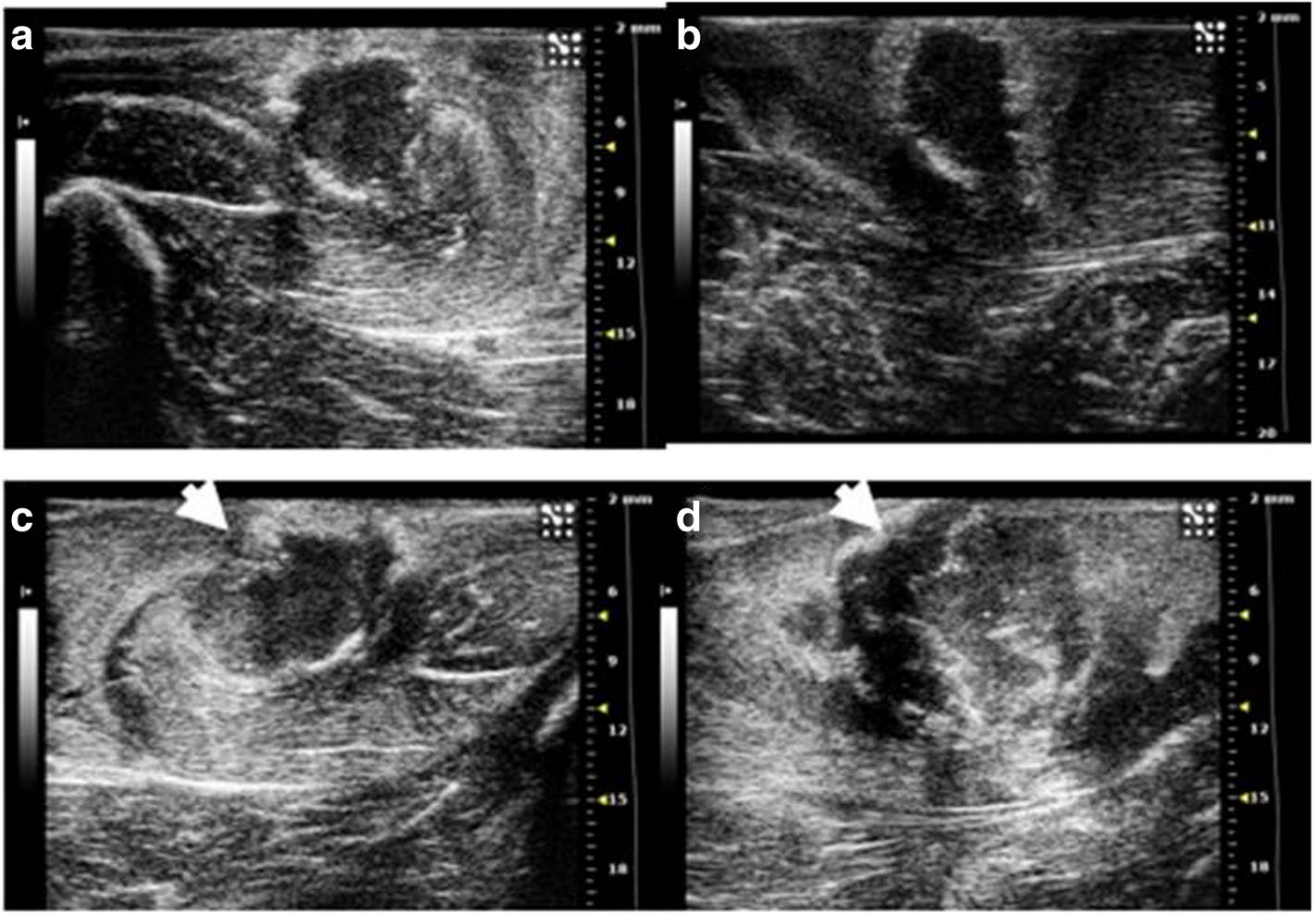
High resolution US of rabbit thigh muscle at 7 (**a**-**b**) and 14 days (**c**-**d**) after VX2 tumor pieces implant. **a**-**b** In the context of the muscular tissue two hypoecoic, irregular masses (*arrows*) are evident, their diameters are of **a**: 9 × 4, 2 × 6, 8 mm **b**: 11 × 9, 4 × 6 mm. **c**-**d** Two irregular masses (*arrows*), hypoecoic, strongly inhomogeneous, are evident. They are increased in size, measuring: **c**: 7,2 × 5, 7 × 8,6 mm; **d**: 5,5 × 12, 6 × 7,3 mm

**Fig. 3:**
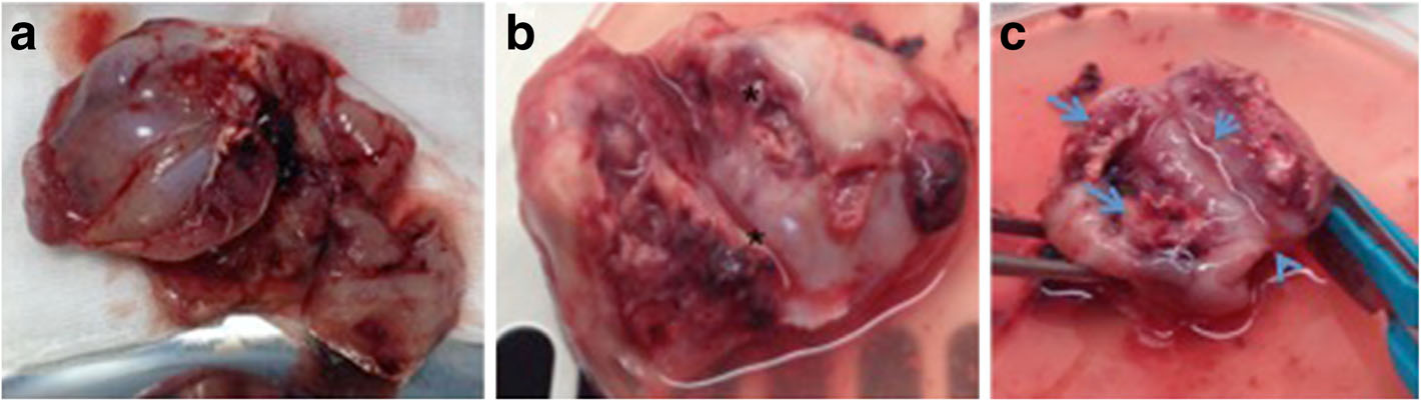
Hind limb VX2 tumor propagation. **a** Pictures reveales explanted hind limb tumor. **b** Tumor excited shows necrotic core (*asterisk*). **c** peripheral viable tumor (*arrowheads*), and adherent muscle tissue (*arrows*)

**Fig. 4:**
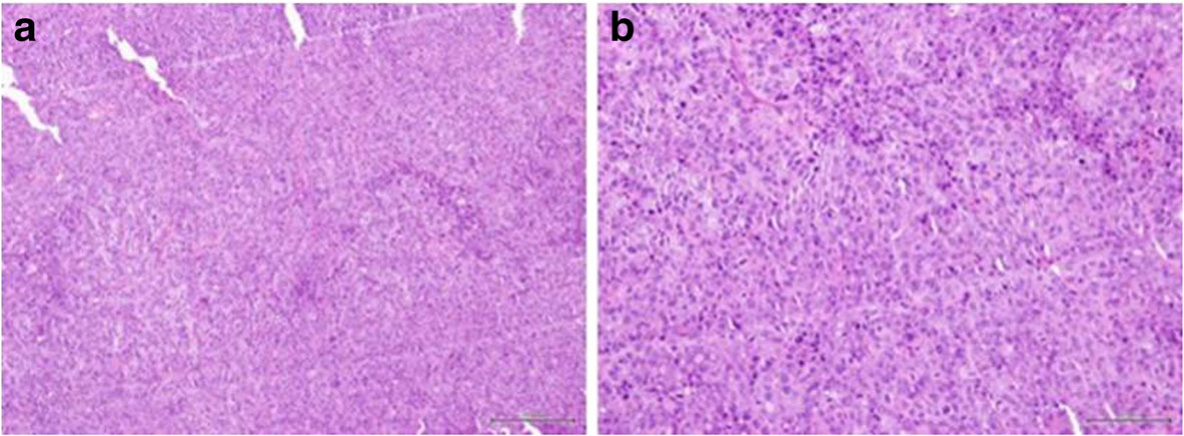
Histological analysis of hindlimb tumors after 14 days from implant. **a**-**b** Positive tumor cells were detected hindlimb tumor tissue without presence of necrotic area. Magnifications: 10 X (**a**) and 20X (**b**)

Four rabbits were used as donors for development of VX2 tumor in the lower para-spinal right muscle of each rabbit’s hind limb. Two small tumor frozen pieces (previously thawed in lukewarm water), were implanted into the quadriceps muscle of each rabbit’s hind limb. A viable tumor of about 2,5 cm × 3 cm was obtained after 10–14 days of tumor growth in each rabbit (Fig. 1). Tumor development and progression was confirmed and monitored by high resolution US performed at 7 and 14 days after surgery, (Fig. 2a-d) detecting tumor as a predominantly hypoecoic, inhomogeneous, irregular, infiltrating mass, growing in the muscular tissue. In order to obtain the propagation of VX2 tumor line and para-renal tumor creation, we performed hind limb tumor extraction after the sacrifice of rabbits. The viable tumors (Fig. 3a-c) were accurately treated as described above. Half of tumor tissues were employed fresh to develop the para-renal rabbit tumor model, while the remaining part was frozen to create a stock. The presence of tumor cells in tumor samples was confirmed by histological analysis (Fig. 4a-b).

### Generation of VX2 para-renal tumor rabbit model

**Fig. 5:**
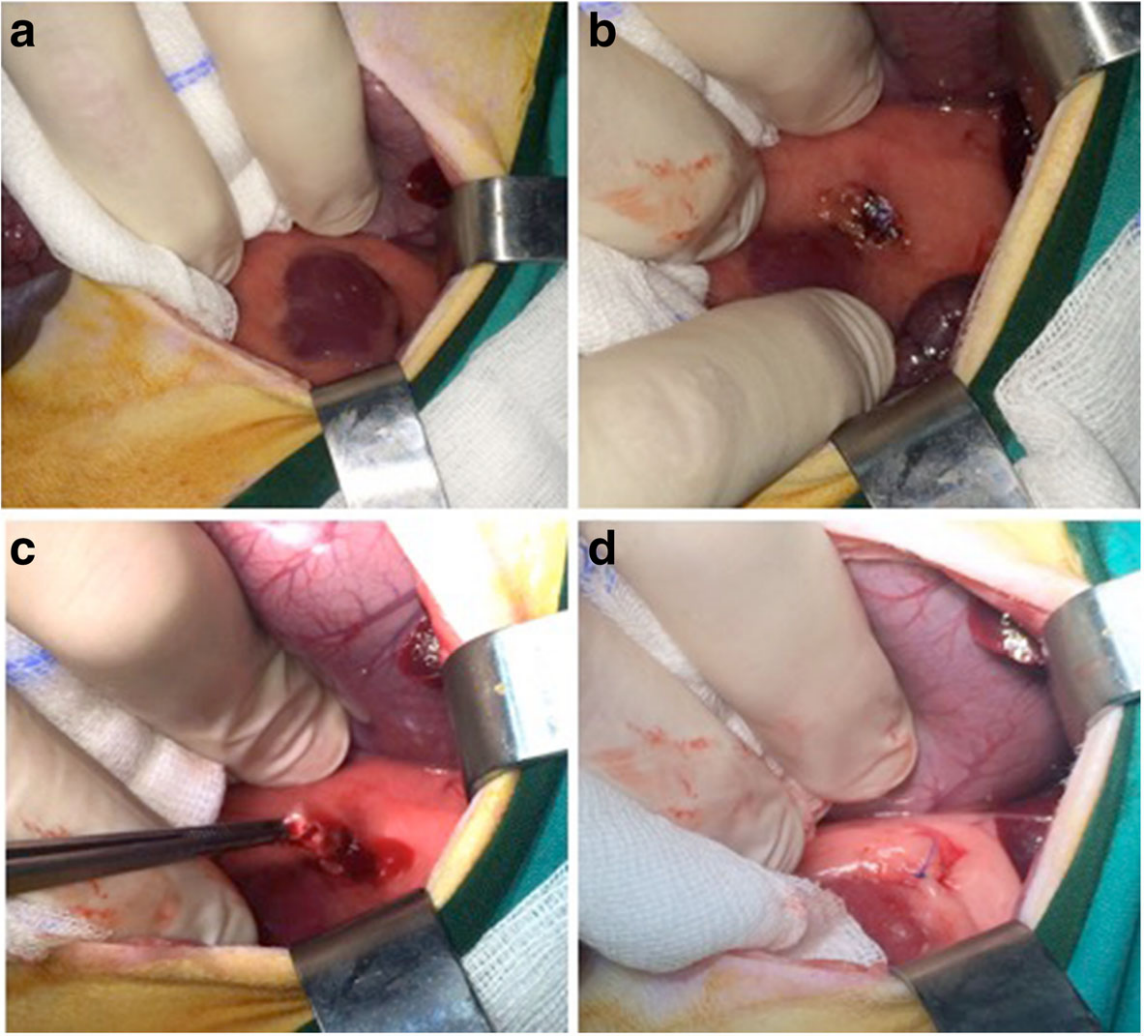
Surgical implantation of VX2 tumor in para-renal region of rabbits. **a** Picture shows the implantation region. **b** The incision of the left retroperitoneal para-renal space. **c** Surgical implantation of VX2 tumor in para-renal region. **d** The para-renal incision closed with prolene 4/0

**Fig. 6:**
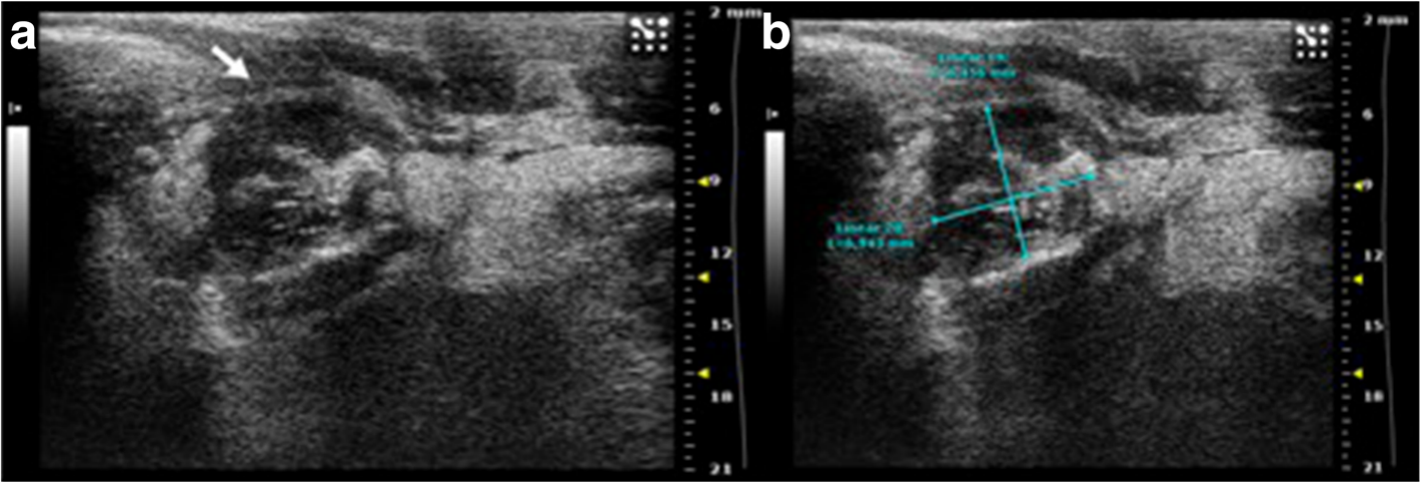
High resolution US in longitudinal plane of the left suprarenal fossa 7 days after the implant of VX2 tumor fragments. An irregular mass, inhomogeneous, hypoecoic is evident above the upper renal pole (*arrow* in **a**) with a diameter of 6,943 × 6,456 mm (**b**)

**Fig. 7:**
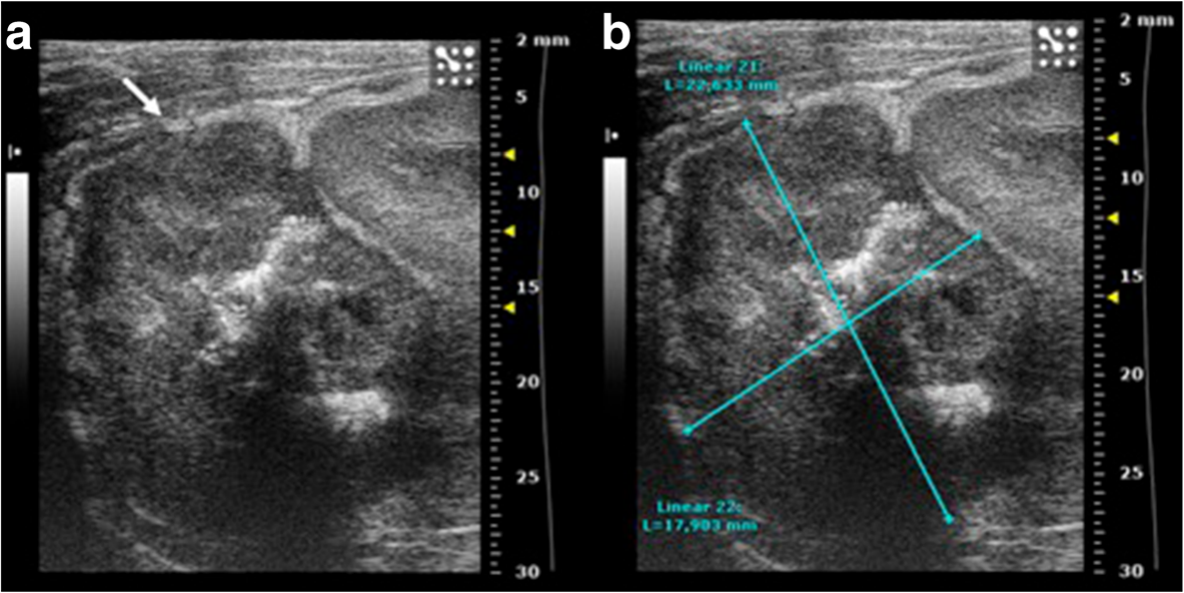
High resolution US in longitudinal plane of the left suprarenal fossa 14 days after the implant of VX2 tumor fragments. The inhomogeneous and hypoecoic mass detected in the upper renal pole increased in size (*arrow* in **a**) with a diameter of mass 17,903 × 22,633 mm (**b**)

**Fig. 8:**
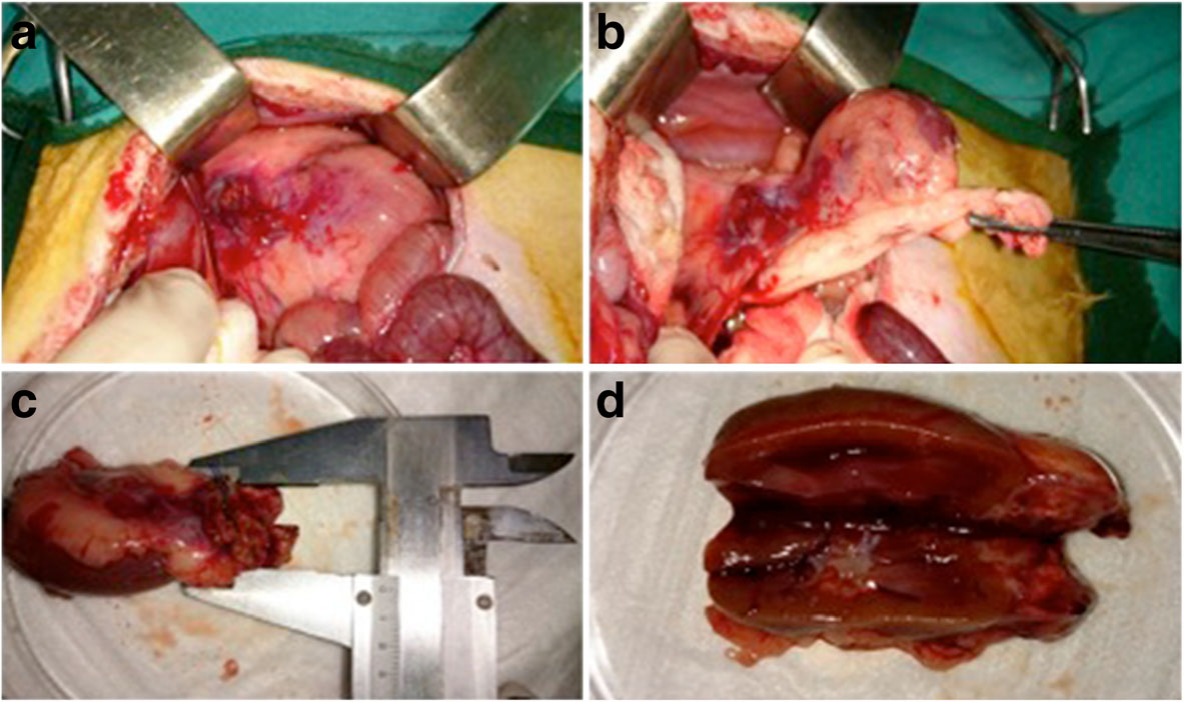
VX2 para-renal tumor in rabbit at 14 days from tumor piece implant. **a**-**b** The images show the surgical procedure for removal of the left kidney and para-renal tumor in rabbit after 14 days from the implant of the VX2 tumor. **c**-**d** The measurement of the tumor, with the caliper, in the para-renal area, reveals the presence of a 1,5 × 1,5 cm nodule. **d** Dissection of left kidney and tumor mass

**Fig. 9:**
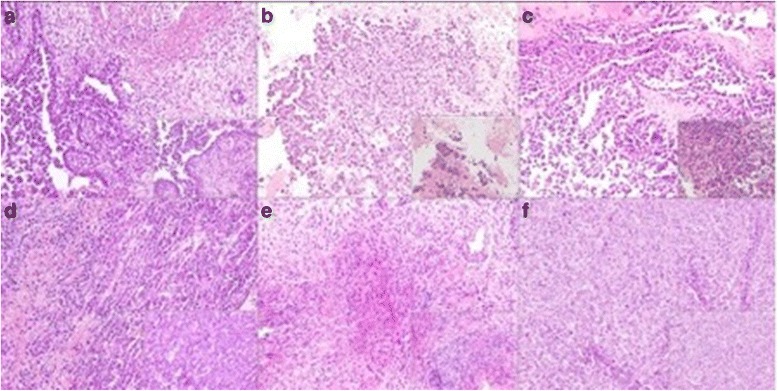
Analysis of histological sections of Vx2 tumor after 7, 14 days from tumor tissue implant. **a**, **b**, **c** The histological analysis of the tumor after 7 days from tumor tissue implant, reveals the presence of active tumor cells and percentage of 5% of necrotic area. **d**, **e**, **f** The histological analysis of the tumor after 14 days from tumor tissue implant, reveals the presence of active tumor cells and percentage of 10% of necrotic area. Magnifications: 20x and 40X (*boxes*)

In order to generate VX2 para-renal tumor rabbit model, the recipient rabbits underwent a xipho-umbilical laparotomy. A blunt dissection was used to expose the peritoneum through the avascular linea alba. Careful division of the peritoneum allows exploring the peritoneal cavity. Intestinal mobilization was performed and an incision of the left retroperitoneal space supra renal capsule was made. A single tumor fragment was then placed in this para-renal space. A single tumor fragment was then placed in para-renal region as showed in Fig. 5. The complete surgical procedure was performed as previously described. Tumor growth was assessed by ultrasound high resolution US performed at 7 and 14 days after surgery, it allowed detection of the tumors as an irregular and inhomogeneous mass, hypoecoic, infiltrating the surrounding tissue, as shown in Figs. 6 and 7 (a-b) respectively. Two weeks after the tumors implantation, rabbits were sacrificed and tumors and other organs, such liver, lung and both kidneys were collected for histopathology analysis (Fig. 8) (Fig. 9 and data not shown). The histological analysis of the tumor after 7 days from tumor tissue implant, revealed the presence of active tumor cells and percentage of 5% of necrotic area. (Fig. 9a, b, c) The histological analysis of the tumor after 14 days from tumor tissue implant, revealed the presence of active tumor cells and percentage of 10% of necrotic area (Fig. 9d, e, f).

It is important to underline that the implantation of tumor fragments achieved tumor growth in 100% of case. To date this is the first report describing a rabbit model of para-renal cancer by surgical tumor implantation of frozen VX2 tumors.

## Discussion

We generated a rabbit model of para-renal cancer by surgical tumor implantation of VX2 frozen tumors into rabbits para-renal region. The VX2 tumor is a leporine anaplastic squamous cell carcinoma virus-induced, and is characterized by rapid growth and hypervascularity. The generation of this cancer model will minimize the development of metastases and the use of non-necrotic tumors. Moreover, with this method, the evaluation of tumor response to loco-regional therapy experiments will be optimized. Various forms of multimodal neoadjuvant and adjuvant treatments have been used in combination with surgery to improve loco regional control of solid tumors having a high incidence of local recurrence after resection, such as rectal cancer [17–21], soft tissue sarcomas [22] and pancreatic cancer [23, 24].

Our animal model can be used in the future experiments, to test the safety and the efficacy, in the bed of resection, of new medical-devices in the multimodal treatment approach of solid tumors with high risk of local recurrence after surgery.

## Conclusions

We generated for the first time, a model of para-renal cancer by surgical tumor implantation of VX2 frozen tumor fragments into rabbit’s para-renal region. This method minimizes the development of metastases and the use of non-necrotic tumors and will optimize the evaluation of tumor response to loco-regional therapy experiments.
